# Age-Associated Changes in Estrogen Receptor Ratios Correlate with Increased Female Susceptibility to Coxsackievirus B3-Induced Myocarditis

**DOI:** 10.3389/fimmu.2017.01585

**Published:** 2017-11-16

**Authors:** Andreas Koenig, Iwona Buskiewicz, Sally A. Huber

**Affiliations:** ^1^Department of Pathology, University of Vermont, Burlington, VT, United States

**Keywords:** aging and immunocompetence, mouse models, T-regulatory cells and innate immunity, estrogen receptor alpha, estrogen receptor alpha:beta ratios and immune competence, sex bias in coxsackievirus B3 myocarditis

## Abstract

Sexual bias is a hallmark in various diseases. This review evaluates sexual dimorphism in clinical and experimental coxsackievirus B3 (CVB3) myocarditis, and how sex bias in the experimental disease changes with increased age. Coxsackieviruses are major causes of viral myocarditis, an inflammation of the heart muscle, which is more frequent and severe in men than women. Young male mice infected with CVB3 develop heart-specific autoimmunity and severe myocarditis. Females infected during estrus (high estradiol) develop T-regulatory cells and when infected during diestrus (low estradiol) develop autoimmunity similar to males. During estrus, protection depends on estrogen receptor alpha (ERα), which promotes type I interferon, activation of natural killer/natural killer T cells and suppressor cell responses. Estrogen receptor beta has opposing effects to ERα and supports pro-inflammatory immunity. However, the sexual dimorphism of the disease is significantly ameliorated in aged animals when old females become as susceptible as males. This correlates to a selective loss of the ERα that is required for immunosuppression. Therefore, sex-associated hormones control susceptibility in the virus-mediated disease, but their impact can alter with the age and physiological stage of the individual.

## Introduction

Myocarditis is an inflammation of the myocardium usually following microbial infections. Most viruses and many bacteria, fungi, protozoa, and helminths can initiate the disease including Picornaviruses and Adenoviruses (1–4). The clinical disease takes multiple forms including acute, chronic, giant cell, and eosinophilic myocarditis with differences in prognosis and in underlying pathogenesis (2, 3). Acute myocarditis may be severe but self-limiting and many investigators hypothesize that cardiac injury results from direct microbial injury to the heart or to antimicrobial host defense mechanisms including cytokine storms, which directly suppress myocyte contractility and function (3, 5, 6). Chronic myocarditis may last for months or years and result in either cardiac transplantation or death. Underlying mechanism for the chronic form remains controversial with dueling theories of persistent virus infection causing myocardial dysfunction or infection triggering autoimmunity to heart antigens (3, 5). Clinical trials of type 1 interferon (IFN) treatment of virus positive cardiomyopathy patients showed virus clearance from the heart and partial improvement of cardiac function through 12 weeks posttreatment although the improvement waned somewhat by 24 weeks posttreatment (7). Lack of a long-lasting protection with virus clearance could circumstantially favor autoimmune pathogenic mechanisms. Immunosuppression improves long-term survival for giant cell myocarditis patients (8) and has also been shown to be effective in acute myocarditis (9). However, not all clinical trials have shown protection with immunosuppression (10). Mouse models of coxsackievirus B3 (CVB3)-induced myocarditis cause cardiac inflammation, which histologically resemble the clinical disease. While virus infection initiates myocarditis, predominant cardiac damage is immune mediated with strong evidence implicating induction of autoimmunity to heart antigens (3). Factors required for autoimmunity include as follows: (1) the existence of autoreactive lymphocytes; (2) autoreactive cell activation; and (3) the absence of immunosuppression (11). Infections can lead to autoimmunity by several mechanisms. These include antigenic mimicry in which microbial and self-molecules share sufficient similarity to induce cross-reactive immunity (12, 13), and such antigenic mimicry has been described between CVB3 and cardiac proteins (14, 15). Another mechanism is an adjuvant effect similar to immunization of self-proteins in complete Freund’s adjuvant (16, 17). The ability of adjuvants to promote autoimmunity probably results from their ability to activate the innate immune system through pathogen-associated molecular patterns stimulation of toll-like receptors (TLR). Giving specific TLR agonists along with cardiac proteins/apoptotic myocytes is sufficient for myocarditis induction while giving either adjuvant or heart tissue alone is ineffective (18, 19). Virus-induced tissue damage releases large amounts of normally sequestered antigens not normally available to the immune system. Autoimmunity can result from cryptic epitopes (20). Under certain conditions, these cryptic epitopes can become visible to the immune system and initiate pathogenic immune responses. While cryptic epitopes result from naturally occurring protein folding/unfolding or protein–protein interactions, most microbes encode their own proteases, which cleave cellular as well as microbial proteins and might produce peptides distinct from those produced by cellular proteases (21).

## Sex-Associated Hormones Control CVB3-Induced Myocarditis Susceptibility

Both the incidence and severity of myocarditis and dilated cardiomyopathy are greater in men than women (22). This is true of enterovirus infections generally (23). CVB3 mouse models of myocarditis infecting young adult animals mimic the male dominance in disease susceptibility with females being largely protected from cardiac inflammation, myocardial injury, and death subsequent to viral infection (22, 24, 25). Testosterone promotes susceptibility as castrated males are protected while exogenous administration of testosterone restores cardiac inflammation. Similarly, estrogen (E2) is protective as ovariectomized females show increased myocarditis compared with intact females and E2 treatment of males is protective (24, 26). E2 levels determine myocarditis resistance since CVB3 infection of females during diestrus when E2 levels are lowest results in myocarditis susceptibility whereas CVB3 infection during estrus is completely protective (27). Sex hormones are well known for their ability to influence innate and adaptive immunity including type 1 IFN response, TLR type and level of activation, antigen presentation [macrophage/dendritic cells (DCs)], and T lymphocyte polarization [reviewed in Ref. (28–33)]. Male and female CVB3-infected mice generate distinct T cell responses to infection with males generating pro-inflammatory and females generating anti-inflammatory or immunosuppressive (T-regulatory) responses (34, 35). Studies by Xiong and colleagues have shown hormonally regulated polarization of macrophage leading to monocytic myeloid suppressor cells in females, and this may result from differences in innate immune responses (cytokine response by natural killer cells) to infection between males and females (36–38).

Mechanisms of estrogen signaling in cells have been extensively reviewed in the literature (33, 39–43) and will only be briefly discussed here. Estrogen mediates its effects primarily through two receptors, such as estrogen receptor alpha (ERα) and estrogen receptor beta (ERβ), which bind the hormone with similar affinity but due to slight differences in the ligand binding pockets of the receptors, may promote transcription of distinct sets of genes (40, 43). Most lymphoid cells express either or both types of estrogen receptors [Table 1; reviewed in Ref. (33, 43, 44)]. The estrogen receptor binds to estrogen in the cytosol forming a complex and translocates to the nucleus for direct interaction with estrogen response elements (ERE) in many gene promoters. Various ERE may differ in sequence resulting in differences in estrogen receptor binding affinity and variation in transcriptional activity. Even the same ERE can produce different transcriptional activation between distinct cell types because of variations in specific factors such as coactivators (40). Although ERβ has many similar characteristics to ERα, molecular mechanisms of transcriptional activation may differ between the receptors (45). Alternatively, the estrogen receptors may initiate transcription without directly interacting with DNA through binding to specific transcription factors including NF-κB, SP1, AP-1, or C/EPBβ, which alters their DNA binding or coregulatory factor complex formation and gene activation (46, 47). In studies evaluating ERα–AP-1 and ERβ–AP-1 response elements, evidence demonstrated that these nuclear hormone receptor subtypes can exert opposite transcription controls on gene expression (48). Thus, in certain situations, ERβ can inhibit ERα-induced gene expression (49). Membrane bound ERα/ERβ, which are splice variants of the nuclear forms, and a transmembrane G protein-coupled estrogen receptor (GPER) induce much more rapid signaling compared with the classical nuclear ER isoforms (41, 42).

**Table 1:** Estrogen receptor expression by cells of the immune system.

Cell	ERα	ERβ	GPER
CD4 T lymphocyte	H (high)	L	H
CD8 T lymphocyte	L (low)	L	
T regulatory cell	L	H	H
B lymphocyte	L	H	H
Natural killer cell	H	H	
Monocyte	L	H	
Macrophage	H	L	H
Myeloid dendritic cell	H	H	

*Yakimchuk et al. (43); Kovats (33); Revankar et al. (41); Soltysik and Czekaj (42); and Klinge (40)*.

*ERα, estrogen receptor alpha; ERβ, estrogen receptor beta; GPER, G protein-coupled estrogen receptor*.

Studies determined that protection during experimental CVB3-induced myocarditis depends on signaling through ERα as disease susceptibility is increased in ERα knockout mice while infected male mice treated with the specific ERα agonist, propylpyrazoletriol (PPT) were protected (50). By contrast, signaling through ERβ promotes myocarditis as shown by treating female or male mice with the specific ERβ agonist, diarylpropionitrile. No studies were found on GPER in myocarditis, but GPER abrogates cardiac myocyte apoptosis during ischemia/reperfusion injury (51) suggesting a cardioprotective role. The mechanisms of ERα-induced protection are likely to be multifaceted. ERα should increase expression of type 1 IFNs (30), which are crucial to preventing cardiac injury (52). Previous studies have shown that E2 promotes T-regulatory cell activation both through upregulation of FoxP3 and through increased T-regulatory cell proliferation (53, 54). In systemic sclerosis patients, FoxP3(bright) cells were increased in patients with autoantibodies to ERα (55). GPER also attenuates autoimmune diseases and elicits FoxP3 expression (56). In the myocarditis model, infecting young female during estrus but not during diestrus, activates T-regulatory cells (57) as does treating males with the ERα agonist, PPT (50). Signaling through the ERβ prevents T-regulatory cell activation. Unlike prior reports, however, control of T-regulatory cell response is not mediated through direct action on FoxP3 transcription but rather through regulation of natural killer T (NKT) and gamma-delta T (γδT) cell responses (50, 58). Various studies report that NKT cell subpopulations suppress autoimmunity and increase T-regulatory cell numbers *in vivo* (59–61). By contrast, γδT selectively kill T-regulatory cells and relieve immunosuppression resulting in autoimmunity (62). While PPT treatment of CVB3-infected males induces T-regulatory cells, this fails to occur in NKT knockout mice demonstrating that the effect of E2 signaling must be indirectly mediated through these innate effectors rather than through modulating FoxP3 expression in T-regulatory precursors directly (50). ERα modulates activities of many TLR most notably TLR 2, 7, 8, and 9 (30, 63, 64). By contrast, ERα may inhibit responses dependent on TLR4 (65). CVB3 infection selectively upregulates TLR2 in females but upregulates TLR4 in males (66, 67). Treating males with the specific TLR2 agonist dramatically reduces mortality while treating females with the TLR4 agonist promotes pathogenicity (19, 66). Interaction of NKT cells with immature myeloid DCs triggers tolerogenic maturation of the DC and T-regulatory cell generation while NKT cells interacting with DC in concert with TLR4 stimulation results in pro-inflammatory responses (68).

## Age and Myocarditis Susceptibility: Loss of Sexual Dimorphism

**Figure 1:**
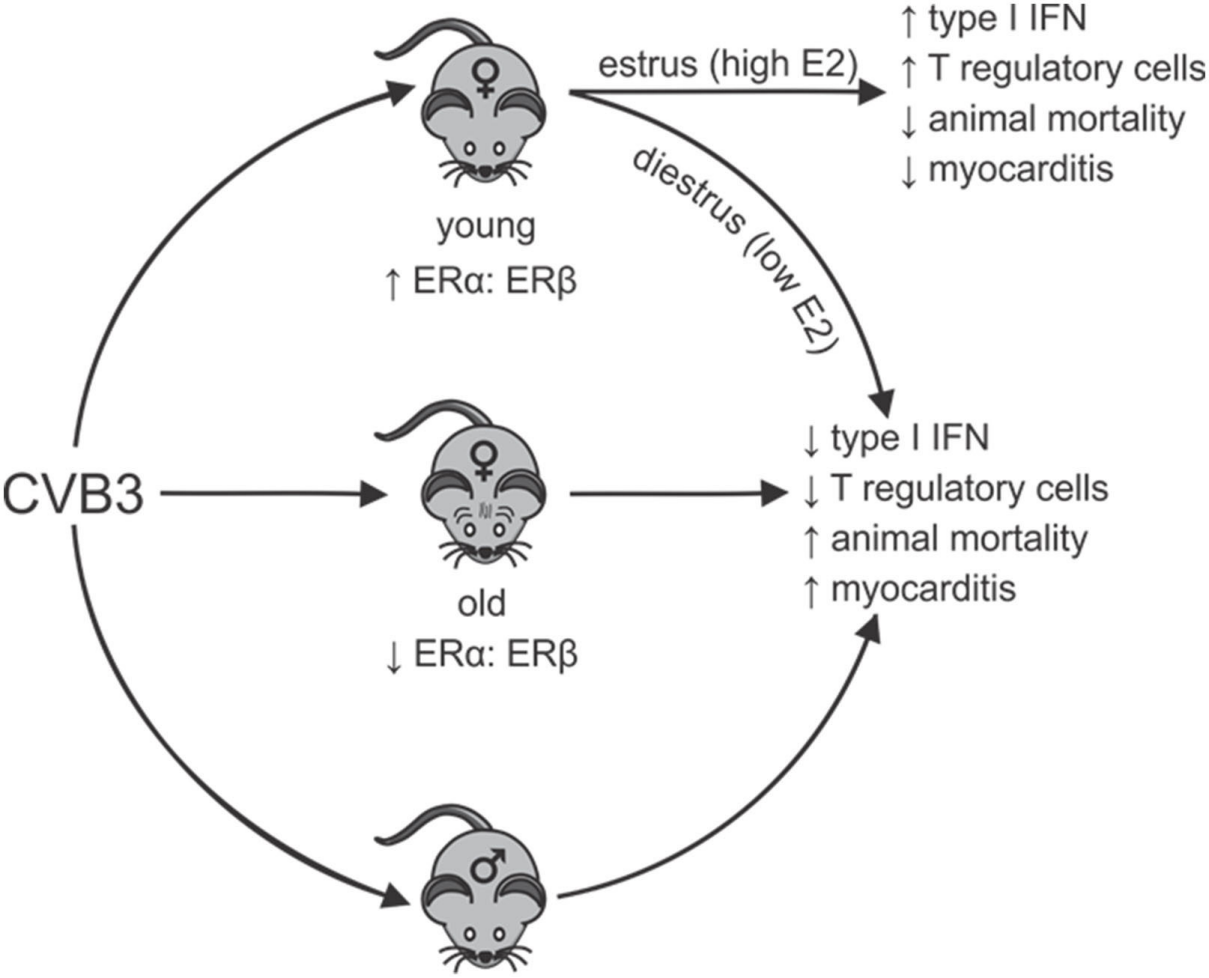
Sexual dimorphism in coxsackievirus B3 (CVB3)-induced myocarditis: dependence on estrogen (E2) concentration and relative estrogen receptor alpha (ERα):estrogen receptor beta (ERβ) expression in lymphoid cells. Young females (age 2–3 months) have high ratios of ERα:ERβ in spleen cells resulting in robust type 1 interferon (IFN) responses to CVB3 infections, especially when virus infection occurs during estrus the time of peak E2. Signaling through ERα promotes T-regulatory cell responses leading to protection from myocarditis and animal mortality. Infection of young females during diestrus when E2 concentrations are low or infection of old females (12 months), which selectively lose ERα but increase ERβ expression in lymphoid cells results in reduced type 1 IFN response to CVB3 and prevents activation of T-regulatory cells. This results in induction of strong pro-inflammatory immunity and increased animal mortality and myocarditis, which is similar to disease observed in males. Thus, there is sexual dimorphism in CVB3-induced disease, but this can be dependent on both the hormonal state of the individual (stage of the estrus cycle when infection occurs) and the age of the individual when infection occurs. The age of the individual could be important as E2 regulation of dendritic cell development is well documented and is ERα dependent. Selective loss of ERα has been shown in the cardiovascular system with age and similar loss in lymphoid cells could affect immunocompetence.

A systemic review of the world literature in 2010 reported no significant difference in age between men and women when evaluating the proportion of heart failure patients caused by myocarditis (69). However, in a report by Laufer-Perl et al. (70) of 200 pericarditis/myocarditis patients, there was both an increased incidence and younger age for men (73%; 46 ± 19 years of age) compared with women (27%; 60 ± 19 years of age). In this latter study, the women had a lower rate of hospitalization raising the possibility that reduced disease severity in women might partially mask some epidemiological characteristics in this sex missed by evaluating heart failure. Also, when combining data encompassing all forms and etiological causes of myocarditis, distinct pathological mechanisms in each type of myocarditis may be impacted by sex-associated hormones differently. In the experimental CVB3 myocarditis model, the clear sexual dimorphism noted in young (2–4 months old) mice is lost in aged (8–12 months old) female mice, which show high mortality, increased cardiac inflammation and a pro-inflammatory phenotype resembling males (71). While exogenous E2 treatment of young females is protective (57), E2 treatment of old females increased both mortality (68.8% or 11/16 mice) and cardiac inflammation (72). Cardiac virus titers increase corresponding with increases in cardiac inflammation, myocyte injury, and animal mortality. Thus, E2 has the opposite effect in young and old female mice during CVB3-induced myocarditis (Figure 1). When spleens of old (12-month old) female mice were analyzed using Western blot and confirmed by RT-PCR, an approximately 90% reduction in ERα and a twofold increase in ERβ was observed compared with young (2.5-month old) female mice, giving a substantially higher ERα:ERβ ratio of in young than in old females. The trend in males was similar but more modest. A reduced ERα in old females corresponds to abrogated T-regulatory cell activation in these animals (50).

## Aging and the Immune System

Aging decreases adaptive immune responses to infection, reduces naïve T cells entering the periphery from the thymus due to thymic involution, and is associated with chronic immune activation and memory cell expansion ultimately leading to immunosenescence and skewering of the immune repertoire at the expense of the ability to respond to new antigens (73–76). Aging affects multiple cells including T cells, B cells, NK cells, DCs, macrophage, neutrophils, and hematopoietic stem cells. DCs are especially important in antigen presentation and ERα but not ERβ has been shown to promote optimal DC differentiation and cytokine production (77). Studies on the effects of aging on DCs have been controversial with some studies reporting no changes in numbers, distribution, turnover, or engraftment ability of aged cells compared with cells from young mice (78); while other studies have found reductions in cDC but not pDC (79). In aged humans, pDC reportedly show both numerical and functional decline in peripheral blood but myeloid DC do not (80). A number of studies report that aged DC from both humans and mice produce less type 1 and type 3 IFNs than cells from young individuals in response to TLR stimulation (81). While divergent reports exist, various studies have found that aging reduces MHC II and co-stimulatory molecule (CD40/CD86), impairs naïve T cell priming and potentially increases the negative co-stimulatory molecule PD-L1, which promotes T-regulatory cell activation (81, 82). Studies in 26-month-old rats show that E2 treatment inhibits maturation and functional activity of DC (83). Aging alters ERα and ERβ expression levels in various tissues most notably in the bone, brain, eye, kidney, and cardiovascular system (84–89). Changes are observed both in humans (84, 86, 90) and in rodents (85, 87, 89). The age-related reduction of ERα in heart and kidney results from increased methylation of the promoter due to oxidative stress (89, 90). Whether reactive oxygen species might similarly reduce ERα in DC is unclear.

## Conclusion

Sex-associated hormones determine enterovirus pathogenesis and act through their effects on the innate immune response. This is demonstrated through modulation of type 1 IFN responses, which can impact virus load in infected tissues but also on both the type and level of TLR expression, on the activation of innate effectors including NK, NKT, γδT and macrophage, and on the adaptive immunity including induction of autoimmunity to heart antigens. Androgens promote pro-inflammatory responses. Estrogens promote anti-inflammatory responses at high levels but are pro-inflammatory at low levels. Aged females become more disease susceptible presumably because of the dual effect of lower estradiol and decreased physiological expression of ERα, the protective estrogen receptor that results in a decreased ERα:ERβ ratio. Whether a similar phenomenon occurs in clinical myocarditis is unclear. Few reports fully address age of men and women with myocarditis separately and with consideration of both the various types of myocarditis and the potential etiological mechanisms of each disease form. The acute form presumably resulting from direct virus injury may experience greater benefit from type 1 IFN and hormones promoting this response while chronic myocarditis might have a different pathogenic mechanism.

## Author Contributions

All authors listed, have made a substantial, direct, and intellectual contribution to the work and approved it for publication.

## Conflict of Interest Statement

The authors declare that the research was conducted in the absence of any commercial or financial relationships that could be construed as a potential conflict of interest.
